# Puerarin Improves Diabetic Aorta Injury by Inhibiting NADPH Oxidase-Derived Oxidative Stress in STZ-Induced Diabetic Rats

**DOI:** 10.1155/2016/8541520

**Published:** 2016-01-06

**Authors:** Wenping Li, Wenwen Zhao, Qin Wu, Yuanfu Lu, Jingshan Shi, Xiuping Chen

**Affiliations:** ^1^Key Lab for Pharmacology of Ministry of Education, Department of Pharmacology, Zunyi Medical College, Zunyi 563003, China; ^2^Chengdu Chronic Diseases Hospital, Chengdu 610083, China; ^3^State Key Laboratory of Quality Research in Chinese Medicine, Institute of Chinese Medical Sciences, University of Macau, Macau

## Abstract

*Objective.* Puerarin is a natural flavonoid isolated from the TCM lobed kudzuvine root. This study investigated the effect and mechanisms of puerarin on diabetic aorta in rats.* Methods.* Streptozotocin- (STZ-) induced diabetic rats were administered with puerarin for 3 weeks. Levels of serum insulin (INS), PGE2, endothelin (ET), glycated hemoglobin (GHb), H_2_O_2_, and nitric oxide (NO) in rats were measured by ELISA and colorimetric assay kits. The aortas were stained with H&E. Moreover, the mRNA expression of ICAM-1, LOX-1, NADPH oxidase 2 (NOX2), and NOX4 and the protein expression of ICAM-1, LOX-1, NF-*κ*B p65, E-selectin, NOX2, and NOX4 in aorta tissues were measured by real-time PCR and Western blot, respectively. The localization of ICAM-1, NF-*κ*B p65, NOX2, and NOX4 in the aorta tissues was also determined through immunohistochemistry.* Results.* Puerarin treatment exerted no effect on fasting blood glucose levels but significantly reduced the serum levels of INS, GHb, PGE2, ET, H_2_O_2_, and NO. In addition, puerarin improved the pathological alterations and inhibited the expression of ICAM-1, LOX-1, NOX2, and NOX4 at both mRNA and protein levels. Puerarin also significantly reduced the number of cells showing positive staining for ICAM-1, NOX2, NOX4, and NF-*κ*B p65.* Conclusion.* Puerarin demonstrated protective effect on the STZ-induced diabetic rat aorta. The protective mechanisms may include regulation of NF-*κ*B and inhibition of NOX2 and NOX4 followed by inhibition of cell adhesion molecule expression.

## 1. Introduction

Diabetes, a chronic disease characterized by hyperglycemia, has become a major health crisis worldwide. The global prevalence of diabetes in 382 million people in 2013 is estimated to rise to 592 million by 2035 [1]. Diabetes leads to an array of chronic microvascular (retinopathy, nephropathy, and neuropathy) and macrovascular (atherosclerosis, ischemic heart disease, stroke, and peripheral vascular disease) complications. These chronic complications are the major causes of the reduced quality of life among diabetics, increased burden to the health care system, and increased diabetes-related mortality [2]. Although the microvascular complications are directly related to the severity and duration of hyperglycemia, the macrovascular complications are the primary causes of mortality, with myocardial infarction and stroke accounting for 80% of all deaths among T2DM patients [3]. Therefore, inhibiting and alleviating the macrovascular complications have become a major challenge in diabetes treatment.

The vascular functions are tightly regulated by a series of vasoactive agents such as nitric oxide (NO), PGE2, and endothelin (ET) [4]. Furthermore, the adhesion molecules such as intercellular adhesion molecule-1 (ICAM-1) and lectin-like oxidized low-density lipoprotein receptor-1 (LOX-1) play important roles in endothelial dysfunction and vascular injury [5]. In addition, the NADPH oxidase (NOX family), one of the main sources of reactive oxygen species (ROS) in vascular, and NF-*κ*B, the key transcription factor in regulating adhesion molecular expression, play important roles in diabetic vascular complications [6, 7].

Puerarin is a natural, flavonoid-rich component of the Chinese herb lobed kudzuvine root. Previous findings showed that puerarin contains multiple bioactive compounds and has been widely used to treat cardiovascular and cerebrovascular diseases, osteonecrosis, Parkinson's disease, Alzheimer's disease, endometriosis, osteoporosis, liver injury, inflammation, and cancer [8, 9]. Studies also showed that puerarin improves diabetic complications by reducing blood glucose [10] and enhancing glucose uptake [11], thereby preventing retinopathy [12], improving insulin resistance (IR) [13], protecting the pancreatic beta cells [14], improving cardiac function [15], and inhibiting oxidative stress [16], among others. However, its effect on diabetic macrovascular complications remains unclear. In the current research, the effect of puerarin on macrovascular complications was investigated in streptozotocin- (STZ-) induced diabetic rats.

## 2. Methods

### 2.1. Reagents

Puerarin injection was purchased from Hunan WZT Pharmaceutical Co., Ltd. (China). The STZ was obtained from Sigma (USA). The primers for ICAM-1, LOX-1, NADPH oxidase 2 (NOX2), NOX4, and *β*-actin were purchased from Generay, Inc. (Shanghai, China). The primary antibodies for ICAM-1, LOX-1, and NF-*κ*B p65 were purchased from Proteintech Group, Inc. (Wuhan, China), Abcam (USA), and Boster (Wuhan, China), respectively, whereas those for NOX2, NOX4, and E-selectin were purchased from Santa Cruz Biotechnology.

### 2.2. Animals

Male Sprague Dawley rats (250–280 g) were purchased from the Experimental Animal Center of Daping Hospital. This study was approved by the Animal Ethics Committee of Zunyi Medical College. All rats were maintained on a 12 h alternating light/dark cycle at 22 ± 2°C and 55%–60% humidity. Diabetes was induced by intraperitoneal (i.p.) injection of freshly prepared STZ in citrate buffer (dissolved in 0.1 mmol/L citrate buffer, pH 4.2–4.5) at a dosage of 60 mg/kg/day for 3 consecutive days. Eight nondiabetic control rats received an equal volume of citric buffer only. Rats with blood glucose levels of ≥16.7 mmol/L after 72 h administration of STZ were considered diabetic. These diabetic rats (24 rats) were randomly divided into 3 groups (8 each) and were administered with or without puerarin (18 and 45 mg/kg/day i.p.) for 3 weeks. Body weight and blood glucose levels were measured twice a week.

### 2.3. Determination of Fasting Blood Glucose (FBG)

FBG was determined twice a week by using ONETOUCH Ultra Glucometer (Johnson & Johnson, USA) in accordance with the manufacturer's instructions.

### 2.4. Determination of Serum Insulin (INS), Glycated Hemoglobin (GHb), PGE2, ET, H_2_O_2_, and NO Levels

Serum levels of INS, GHb, PGE2, ET, H_2_O_2_, and NO were determined using commercial ELISA kits (R&D, USA) in accordance with the manufacturer's instructions.

### 2.5. H&E Staining

Aorta specimens were fixed in 4% neutral formaldehyde solution. After dehydration with graded alcohol solutions and xylene, the specimens were embedded in paraffin. The specimens were then cut into 5 *μ*m thick cross sections and stained with H&E by conventional method.

### 2.6. RT-PCR

Total RNA was extracted from the aorta specimens with Trizol (Invitrogen) for cDNA synthesis by using a reverse transcription reaction kit (TaKaRa).  Table 1 shows the primers for ICAM-1, LOX-1, NOX2, NOX4, and *β*-actin.

### 2.7. Western Blot

Total proteins were extracted from the aorta, and the protein contents were determined using a BCA Protein Assay Kit (Generay). Proteins (50 *μ*g) were subjected to 8%–10% SDS-PAGE and then transferred onto PVDF membranes. After blocking with 5% nonfat milk in TBST at room temperature for 1 h, the membranes were washed thrice with TBST and then incubated overnight with specific primary antibodies (1 : 500–1 : 1000) at 4°C. After washing with 5% nonfat milk/TBST, the membranes were incubated with horseradish peroxidase-conjugated secondary antibodies at room temperature for 2 h. Protein-antibody complexes were detected by ECL Advanced Western Blot Detection Kit.

### 2.8. Immunohistochemistry

After the consecutive steps of deparaffinization, deactivation of endogenous peroxidase with 3% H_2_O_2_, and antigen blocking with 5% BSA-PBS, the sections were incubated with ICAM-1, NF-*κ*B, NOX2, and NOX4 antibodies (1 : 50 dilution) at 37°C for 2 h. After rinsing thrice with PBS, the sections were incubated with secondary antibody (Gene Tech, Shanghai, China) for 30 min at 37°C and then stained using DAB chromogen kit (Beijing Zhongshan Golden Bridge Biotechnology Co., Ltd.).

### 2.9. Statistical Analysis

Data were expressed as means ± SD of at least three separate experiments. Statistical analysis was performed using SPSS 16.0, and differences between groups were analyzed by one-way ANOVA. A value of *p* < 0.05 was considered statistically significant.

## 3. Results

### 3.1. Effect of Puerarin on Body Weight and FBG

**Figure 1:**
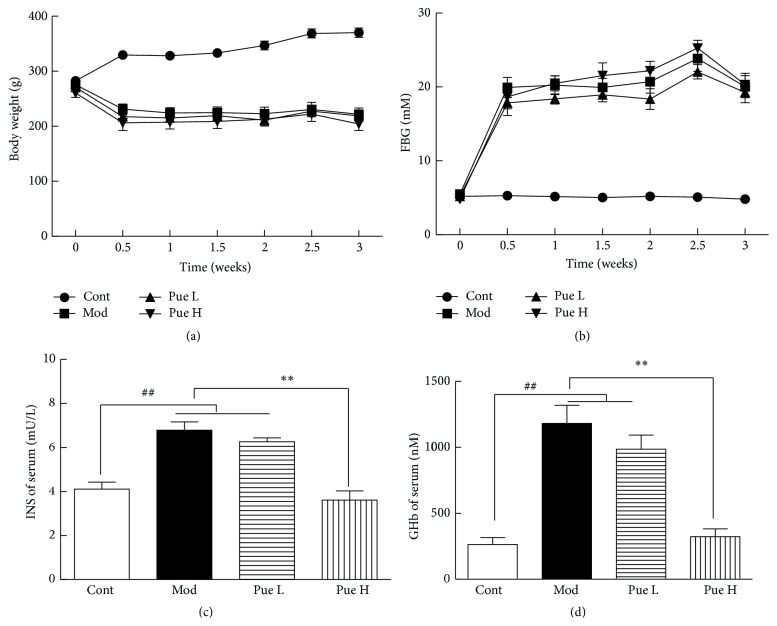
Effect of puerarin on body weight (a), FBG (b), serum INS (c), and GHb in STZ-induced diabetic rats. ^##^ *p* < 0.01 versus Cont; ^*∗∗*^ *p* < 0.01 versus Mod. Cont, control group; Mod, diabetic model group; Pue L, low dosage of puerarin; Pue H, high dosage of puerarin.

The average body weight of the control group increased significantly, whereas that of the model group decreased considerably. Puerarin treatment exerted no effect on body weight (Figure 1(a)). Moreover, the mean FBG of the control group was 5.1 mmol/L, which increased to 20.8 mmol/L after STZ injection approximately fourfold. Compared with the model group, the FBG levels of the high-dosage treatment group decreased, but this disparity was not statistically significant (Figure 1(b)).

### 3.2. Effect of Puerarin on Serum INS and GHb

Compared with the control group, the serum levels of INS and GHb in the diabetic rats increased significantly. Low-dose puerarin exerted no effect on either INS or GHb, whereas high-dose puerarin significantly reduced both INS and GHb levels (Figures 1(c) and 1(d)).

### 3.3. Effects of Puerarin on Serum PGE2, ET, H_2_O_2_, and NO

**Figure 2:**
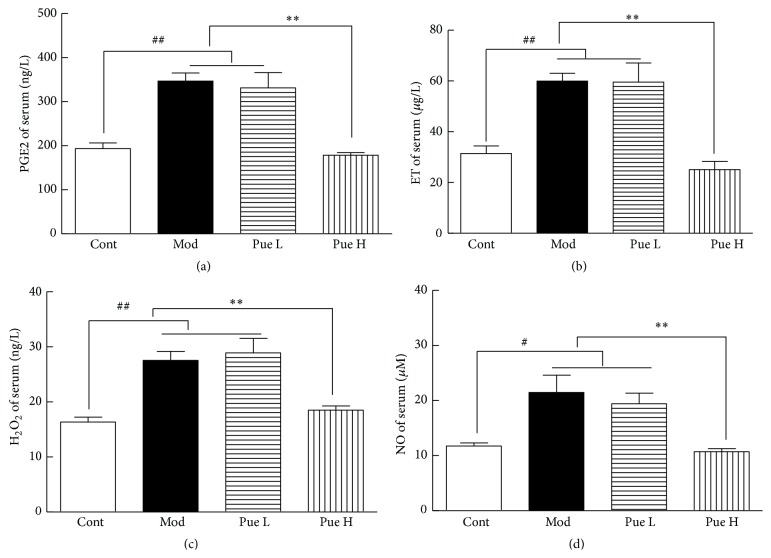
Effect of puerarin on serum PGE2 (a), ET (b), H_2_O_2 _(c), and NO (d) in STZ-induced diabetic rats. ^##^ *p* < 0.01 versus Cont; ^*∗∗*^ *p* < 0.01 versus Mod. Cont, control group; Mod, diabetic model group; Pue L, low dosage of puerarin; Pue H, high dosage of puerarin.

Compared with the control group, the serum levels of PGE2, ET, H_2_O_2_, and NO in diabetic rats significantly increased. These parameters were not affected by low-dose puerarin but significantly reduced by high-dose puerarin (Figure 2).

### 3.4. Effect of Puerarin on Aorta Alterations

**Figure 3:**
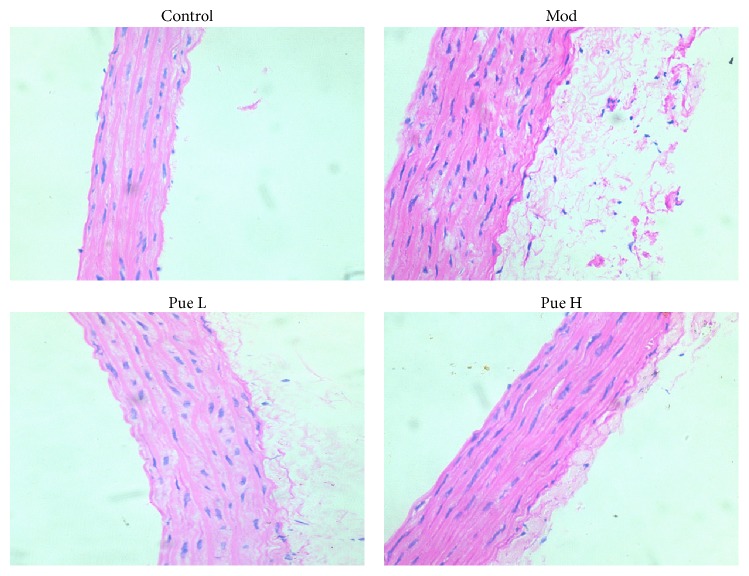
Effect of puerarin on morphological alterations in STZ-induced diabetic rats. H&E staining, 200x. Pue L, low dosage of puerarin; Pue H, high dosage of puerarin.

H&E staining showed that the structure of each layer of the aorta was normal, and the smooth muscle cells were neatly arranged in rows. No damage or injury was observed in the control group, whereas the aortic wall was thickened and the adventitial fibrosis increased in the diabetic group. Furthermore, ruptured smooth muscles and increased nucleus were observed. These alterations were improved by both dosages of puerarin treatment, especially the high-dosage treatment (Figure 3).

### 3.5. Effect of Puerarin on mRNA Expression of ICAM-1, LOX-1, NOX2, and NOX4

**Figure 4:**
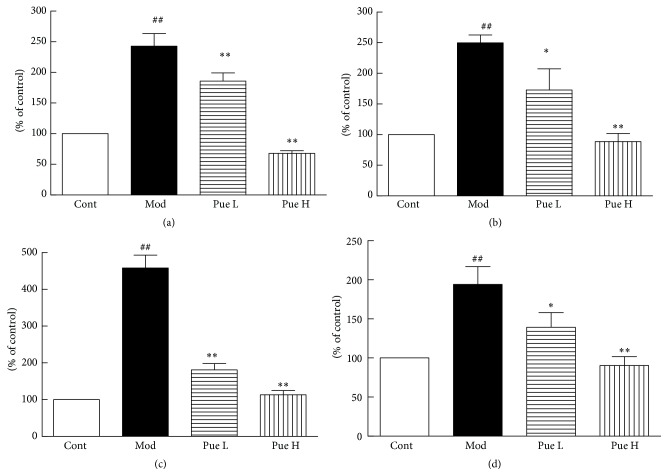
Effect of puerarin on mRNA expression of ICAM-1 (a), LOX-1 (b), NOX2 (c), and NOX4 (d) in the aorta of STZ-induced diabetic rats. ^##^ *p* < 0.01 versus Cont; ^*∗*^ *p* < 0.05 and ^*∗∗*^ *p* < 0.01 versus Mod. Cont, control group; Mod, diabetic model group; Pue L, low dosage of puerarin; Pue H, high dosage of puerarin.

Compared with the control group, the mRNA expression of ICAM-1, LOX-1, NOX2, and NOX4 increased significantly in diabetic rats. The mRNA expression levels of these genes were inhibited by puerarin in a dose-dependent manner (Figure 4).

### 3.6. Effect of Puerarin on Protein Expression of ICAM-1, LOX-1, NOX2, NOX4, E-Selectin, and NF-*κ*B p65

**Figure 5:**
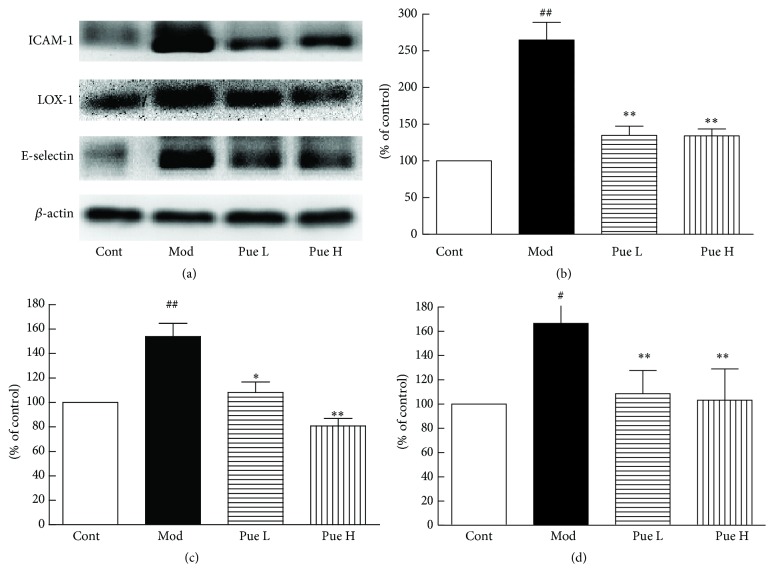
Effect of puerarin on protein expression of ICAM-1, LOX-1, and E-selectin (a) in the aorta of STZ-induced diabetic rats; statistical results for ICAM-1 (b), LOX-1 (c), and E-selectin (d). ^#^ *p* < 0.05 and ^##^ *p* < 0.01 versus Cont; ^*∗*^ *p* < 0.05 and ^*∗∗*^ *p* < 0.01 versus Mod. Cont, control group; Mod, diabetic model group; Pue L, low dosage of puerarin; Pue H, high dosage of puerarin.

**Figure 6:**
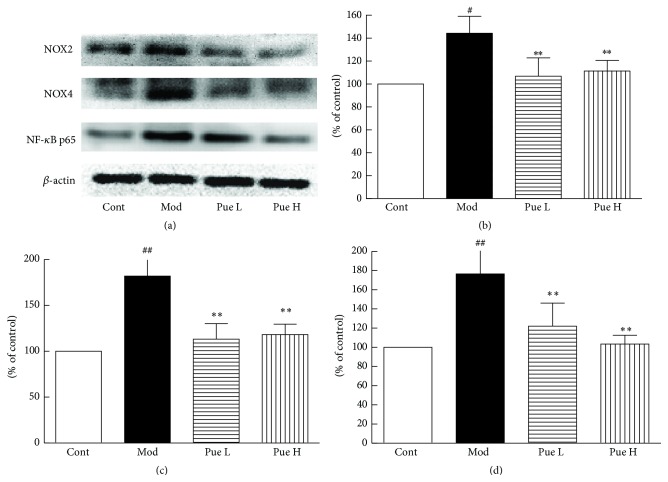
Effect of puerarin on protein expression of NOX2, NOX4, and NF-*κ*B p65 (a) in the aorta of STZ-induced diabetic rats; statistical results for NOX2 (b), NOX4 (c), and NF-*κ*B p65 (d). ^#^ *p* < 0.05 and ^##^ *p* < 0.01 versus Cont; ^*∗*^ *p* < 0.05 and ^*∗∗*^ *p* < 0.01 versus Mod. Cont, control group; Mod, diabetic model group; Pue L, low dosage of puerarin; Pue H, high dosage of puerarin.

Compared with the control group, the protein expression levels of ICAM-1, LOX-1, and E-selectin (Figure 5), as well as those of NOX2, NOX4, and NF-*κ*B p65 (Figure 6), increased significantly in diabetic rats and were inhibited by puerarin.

### 3.7. Effect of Puerarin on Localization of ICAM-1, NF-*κ*B p65, NOX2, and NOX4

**Figure 7:**
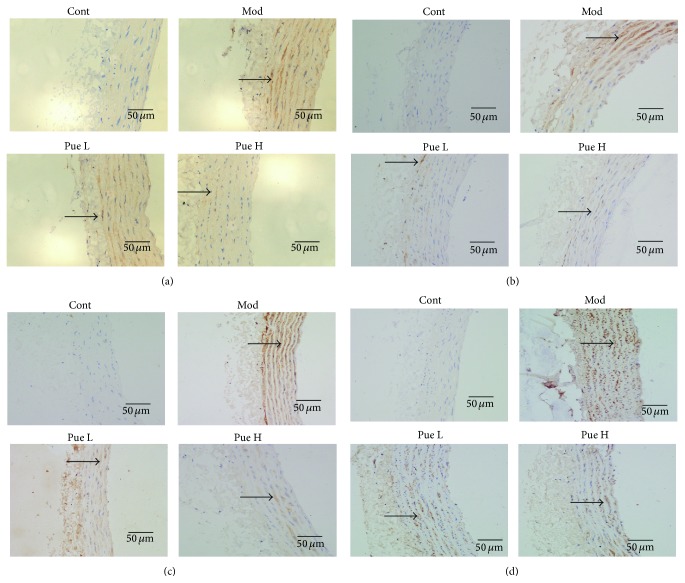
Effect of puerarin on localization of ICAM-1 (a), NOX2 (b), NOX4 (c), and NF-*κ*B p65 (d) proteins in the aorta as revealed by immunohistochemistry. Arrows, intensive expression.

ICAM-1, NOX2, NOX4, and NF-*κ*B p65 were lowly expressed in the endothelial and smooth muscle cells of the control group. By contrast, their expression increased significantly, especially in the smooth muscle cells of the aorta of diabetic rats, as indicated by the conspicuous brown granules. Puerarin treatment significantly reduced the amount of brown granules, suggesting the reduced expression of these proteins (Figure 7). Furthermore, high dosage of puerarin can almost completely inhibit the expression of ICAM-1 and NOX2.

## 4. Discussion

Diabetes, regardless of its clinical categories, is a metabolic disease characterized by high blood glucose level over a prolonged period. The diabetic vascular complications, the main cause of diabetic death, are closely connected with the sustained increase of glucose. In this study, we established a diabetic model with dramatic increase of glucose level, increase of serum INS, and decrease of body weight. Though these characteristics are not identical to classical type 1 or type 2 diabetic rat models, the increased glucose level was useful for the exploration of vascular protective agents.

Puerarin has been approved by the SFDA of China as an adjuvant treatment of coronary heart disease, myocardial infarction, and cerebrovascular disease. The present study investigated the effect of puerarin on the aorta of diabetic rats. STZ injection significantly induced hyperglycemia, increased INS, and reduced body weight, suggesting the development of diabetes. Puerarin exerted no effect on FBG of the STZ-induced diabetic rats, and this finding is consistent with previous report [17]. However, She et al. [16] showed that puerarin treatment reduced blood glucose levels and increased body weight. This disparity may be ascribed to the difference in dosage. Our dosage is less than half of that used by She et al. [16]. Moreover, blood GHb level plays a pivotal role in monitoring the long-term glycemic status of diabetes mellitus patients [18]. In the current study, although puerarin exerted no effect on FBG, puerarin reduced GHb, and this finding is consistent with previous observation on puerarin-treated diabetic patients [19]. Previous research also showed that puerarin reversed the reduced INS in STZ-induced diabetic mice [20]. By contrast, we found that puerarin reversed the increased INS in STZ-induced diabetic rats. This effect of puerarin may be associated with its influence on IR [13]. The contraction and relaxation of vessels are strictly and precisely controlled by vasoactive substances, such as angiotensin II, ET, PGEs, and NO. Consistent with previous reports [21, 22], serum NO and H_2_O_2_ concentrations were significantly increased in diabetic rats. Although physiological levels of NO and H_2_O_2_ positively regulate the vascular function and homeostasis, nitrosative and oxidative stresses caused by excessive generation of them result in alterations of vascular reactivity and lead to vascular toxicity [23]. Increased PGE2 and ET secretions in diabetic vascular bed [24] and placenta [25] were also observed. These changes reflected diabetic vascular dysfunction, which was partly reversed by puerarin, suggesting that puerarin affects diabetic vascular nitrosative and oxidative stresses, and restored vascular tension. Furthermore, pathological alterations were detected in diabetic aorta. The thickened media were mainly caused by the proliferation of vascular smooth muscles, possibly resulting from increased amount of glucose [26]. These observations are consistent with the recent findings in which puerarin attenuates calcification of vascular smooth muscle cells (VSMCs) [27] and attenuates high-glucose- and diabetes-induced VSMC proliferation [28].

The vascular adhesion molecules ICAM-1, LOX-1, and E-selectin serve as biomarkers of vascular inflammation. Enhanced expression of these molecules was observed in high-glucose-treated endothelial cells [29, 30]. In the present study, both the expression of ICAM-1 and LOX-1 at mRNA and protein levels and the protein expression of E-selectin increased in diabetic aorta. Moreover, immunohistochemistry results showed that increased ICAM-1 was observed not only in endothelial cells but also in VSMCs. Puerarin inhibited the expression of these adhesion molecules, suggesting its inhibitory effect on vascular inflammation.

Oxidative stress plays a key role in diabetic complications in the kidney, heart, eye, or vasculature [31]. NOX is a major source of reactive oxygen species (ROS) and is a critical mediator of redox signaling in diabetes [32]. The NOX family comprises seven members, including NOX1–5, DUOX1, and DUOX2. NOX2 and NOX4 are widely expressed in vascular endothelial cells and VSMCs [32, 33]. We found herein a low expression of both NOX2 and NOX4 at mRNA and protein levels in the aorta. Immunohistochemistry results also showed that both endothelial cells and VSMCs expressed NOX2 and NOX4. In addition, their expression was significantly increased in STZ-induced diabetic rats, especially in their VSMCs. These results suggested that NOX was activated in diabetic aorta. Furthermore, superoxide anion and H_2_O_2_ are the main products of NOX2 and NOX4, respectively, and the increased serum H_2_O_2_ concentration is possibly caused by NOX4 activation. Previous studies showed that puerarin inhibits high-glucose-induced VSMC proliferation by interfering with PKC*β*2/Rac1-dependent ROS [28]; moreover, puerarin inhibits retinal pericyte apoptosis induced by the end products of advanced glycation* in vitro* and* in vivo* by inhibiting NOX-related oxidative stress [34]. The inhibitory effect of puerarin on NOX2 and NOX4 expression in diabetic aorta suggested that the beneficial effect of puerarin is attributed to its inhibitory effect on NOX.

ROS is a key upstream activator of the NF-*κ*B pathway [35], which consequently regulates the expression of LOX-1, ICAM-1, and E-selectin [36–39]. The NF-*κ*B pathway was activated as evidenced by the increased protein expression of NF-*κ*B p65 in the diabetic aorta. Furthermore, the expression of NF-*κ*B p65 was colocalized with those of NOX2, NOX4, and ICAM-1, suggesting the potential role of NF-*κ*B. The inhibitory effect of puerarin on NF-*κ*B is possibly caused by the inhibitory effect of puerarin on NOX2 and NOX4, as well as its antioxidant activities [9].

In summary, this study showed that puerarin protected the diabetic aorta by inhibiting oxidative stress and adhesion molecule expression. This finding proves that puerarin can improve the macrovascular complications of diabetes.

## Figures and Tables

**Table 1:** Primers for RT-PCR.

Gene	Forward primer (5′-3′)	Reverse primer (5′-3′)
NOX4	CAGTCAAACAGATGGGATACAGA	ATAGAACTGGGTCCACAGCAGA
ICAM-1	GCTCAGGTATCCATCCATCCC	AGTTCGTCTTTCATCCAGTTAGTCT
LOX-1	TAACTGGGAAAAAAGTCGGGAGAAT	AATGGGAAGTTGCTTGTAAGACGAA
NOX2	TGAATCTCAGGCCAATCACTTT	ATGGTCTTGAACTCGTTATCCC
*β*-actin	CTGAACCCTAAGGCCAACCG	GACCGAGGCATACAGGGACAA
